# Rapid Screening for CRISPR-Directed Editing of the *Drosophila* Genome Using *white* Coconversion

**DOI:** 10.1534/g3.116.032557

**Published:** 2016-08-19

**Authors:** Daniel Tianfang Ge, Cindy Tipping, Michael H. Brodsky, Phillip D. Zamore

**Affiliations:** * RNA Therapeutics Institute, University of Massachusetts Medical School, Worcester, Massachusetts 01605; † Interdisciplinary Graduate Program, University of Massachusetts Medical School, Worcester, Massachusetts 01605; ‡ Howard Hughes Medical Institute, University of Massachusetts Medical School, Worcester, Massachusetts 01605; § Department of Molecular, Cell, and Cancer Biology, University of Massachusetts Medical School, Worcester, Massachusetts 01605; ** Department of Biochemistry and Molecular Pharmacology, University of Massachusetts Medical School, Worcester, Massachusetts 01605

**Keywords:** Cas9, coconversion, genetic screen, microhomology-mediated end joining, homologous recombination

## Abstract

Adoption of a streamlined version of the bacterial clustered regular interspersed short palindromic repeat (CRISPR)/Cas9 defense system has accelerated targeted genome engineering. The *Streptococcus pyogenes* Cas9 protein, directed by a simplified, CRISPR-like single-guide RNA, catalyzes a double-stranded DNA break at a specific genomic site; subsequent repair by end joining can introduce mutagenic insertions or deletions, while repair by homologous recombination using an exogenous DNA template can incorporate new sequences at the target locus. However, the efficiency of Cas9-directed mutagenesis is low in *Drosophila melanogaster*. Here, we describe a strategy that reduces the time and effort required to identify flies with targeted genomic changes. The strategy uses editing of the *white* gene, evidenced by altered eye color, to predict successful editing of an unrelated gene-of-interest. The red eyes of wild-type flies are readily distinguished from white-eyed (end-joining-mediated loss of White function) or brown-eyed (recombination-mediated conversion to the *white^coffee^* allele) mutant flies. When single injected G0 flies produce individual G1 broods, flies carrying edits at a gene-of-interest were readily found in broods in which all G1 offspring carried *white* mutations. Thus, visual assessment of eye color substitutes for wholesale PCR screening of large numbers of G1 offspring. We find that end-joining-mediated mutations often show signatures of microhomology-mediated repair and that recombination-based mutations frequently involve donor plasmid integration at the target locus. Finally, we show that gap repair induced by two guide RNAs more reliably converts the intervening target sequence, whereas the use of *Lig4^169^* mutants to suppress end joining does not improve recombination efficacy.

The ability to make targeted changes in the genome of virtually any organism is transforming biological research. Early genome editing strategies used zinc-finger nucleases (Kim *et al.* 1996; Smith *et al.* 1999; Bibikova *et al.* 2001) or transcription activator-like effector nucleases (Boch *et al.* 2009; Moscou and Bogdanove 2009; Christian *et al.* 2010) that required the construction of unique proteins for each target site. In contrast, the discovery that a chimeric single-guide RNA (sgRNA) can direct the *Streptococcus pyogenes* type II clustered regular interspersed short palindromic repeat (CRISPR)-associated protein 9 (Cas9) to catalyze site-specific double-stranded DNA breaks (DSBs) has eliminated laborious protein construction (Jinek *et al.* 2012; Qi *et al.* 2013). To date, Cas9 is active in all tested organisms including bacteria, plants, fungi, and animals (for reviews see Hsu *et al.* 2014; Sander and Joung 2014; Sternberg and Doudna 2015; Govindan and Ramalingam 2016).

DSBs induced by sgRNA-guided Cas9 stimulate host DNA repair pathways. In many cases the breaks are perfectly rejoined, recreating the original target site, which can be cut again. Occasionally, error-prone end joining inserts or deletes nucleotides at the target site thereby preventing recutting. Such insertions, deletions, and substitutions, collectively called indels, can disrupt a protein-coding sequence. When a DNA donor is supplied exogenously, the DSB can be repaired by homologous recombination (HR), allowing the incorporation of novel sequences at the target site. Unlike sequences incorporated via transgenes, modifying an endogenous gene preserves the chromatin context, enhancers, promoters, introns, and post-transcriptional regulatory elements of the wild-type locus.

Cas9-mediated genome editing requires just three components: (1) Cas9, which can be provided as a purified protein, mRNA, or gene; (2) sgRNA, which can be provided as an RNA or transcribed *in vivo* from a DNA template; and (3) a DNA donor bearing the target sequence containing indels or novel sequences to be incorporated. In *Drosophila*, providing Cas9, sgRNA, and donor DNA transgenes efficiently triggers editing, but establishing the requisite fly stocks takes over a month (Kondo and Ueda 2013; Port *et al.* 2014, 2015; Chen *et al.* 2015). Injecting sgRNA and donor DNA into Cas9-expressing embryos requires far less time but is also less efficient, making it necessary to screen large numbers of animals. Cointegrating a visible marker such as GFP into the target locus can speed the identification of recombinants (Baena-Lopez *et al.* 2013; Gratz *et al.* 2014; Port *et al.* 2014, 2015; Ren *et al.* 2014a,b; Yu *et al.* 2014; Zhang *et al.* 2014b; Chen *et al.* 2015). However, removing the GFP marker by site-specific recombination (*e.g.*, Cre-*LoxP*) takes multiple generations, negating the time advantage of injection and leaving a “scar” sequence (*e.g.*, *LoxP*) at the target site. Indels, of course, must be identified molecularly or through complementation analysis.

In *Caenorhabditis elegans*, coconversion strategies targeting a marker gene together with the gene-of-interest speed the screening for indels and recombinants and avoid introducing an exogenous marker gene at the target locus (Arribere *et al.* 2014; Kim *et al.* 2014; Ward 2015). The coconversion strategy restricts molecular screening to marker-positive animals, substantially reducing the work required to find mutant or recombinant animals. In theory, a similar coconversion system should speed genome editing in *Drosophila melanogaster*.

Here, we describe a strategy in which cotargeting the eye-color gene *white* (*w*) speeds identification of both mutants and recombinants at the gene-of-interest. In our strategy, indels generate loss-of-function *w* mutants whose eyes are white, instead of the wild-type red. In contrast, recombination with the exogenous *w^coffee^* (*w^cf^*) donor DNA produces flies with reddish brown eyes. Mating the injected animals to *w^1118^* null flies and examining the eye color of their offspring allows rapid identification of parents that produce only *w^−^* or *w^cf^* gametes. These flies have an enhanced frequency of indels or recombination at the cotargeted gene-of-interest.

While developing this coconversion strategy for fly genome editing, we also discovered that Cas9-induced recombinants frequently harbor undesirable integration of the entire donor plasmid at the target locus. We find that inducing gap repair with a pair of sgRNAs increases the likelihood of conversion of the intervening target region. Moreover, when DSBs are repaired by end joining, the junction site frequently contains microhomologies or templated insertions, suggesting that the Cas9-catalyzed DSBs are repaired by the microhomology-mediated end-joining pathway and not by the canonical Ligase 4 (Lig4)-dependent nonhomologous end joining; injecting into Cas9-expressing *Lig4^169^* mutants to block canonical end joining neither decreases the yield of indels nor increases the yield of recombinants. Our protocol should reduce the time and effort needed to modify specific loci in the *Drosophila* genome, especially when generating Cas9-induced recombinants.

## Materials and Methods

### Fly stocks

*vas-*Cas9 (*y^1^*, *M{vas-Cas9}ZH-2A*) was generated by recombining *y^1^*, *M{vas-Cas9}ZH-2A*, *w^1118^* (Bloomington #51323; Gratz *et al.* 2014) with Oregon-R. *vas-*Cas9, *Lig4^169^* (*y^1^*, *M{vas-Cas9}ZH-2A*, *Lig4^169^*) was generated by recombining *y^1^*, *M{vas-Cas9}ZH-2A* with *w^1118^*, *Lig4^169^* (Bloomington #28877; McVey *et al.* 2004b). Rainbow Transgenic Flies, Inc. (Camarillo, CA) performed injections.

### sgRNA-expressing plasmid construction

#### sgRNA design:

Target loci of the injection strains were sequenced before sgRNAs designed using crispr.mit.edu (Hsu *et al.* 2013). Guides were preferred if nucleotides 19 and 20 were purines (Farboud and Meyer 2015); positions 15–20, the protospacer-adjacent motif-proximal nucleotides, were >33% GC (Ren *et al.* 2014b); and the sequence placed the guide close to the site of modification. Supplemental Material, Table S7 in File S1 lists sgRNA sequences.

#### sgRNA cloning:

pCFD4, which expresses one sgRNA from a *U6:3* promoter and another sgRNA from a *U6:1* promoter (Addgene #49411; Port *et al.* 2014), was modified to remove *vermillion* and *attB* (pCFD4d). Sequence- and ligation-independent cloning (Jeong *et al.* 2012) was used to clone two guides into *Bbs*I-digested pCFD4d following a PCR incorporating one guide after the *U6:1* promoter, and the other after the *U6:3* promoter (Port *et al.* 2014). Table S8 in File S1 lists the PCR primers. The 20 nt *w* sgRNA-2 template was inserted into the *Bbs*I sites of pDCC6, which expresses sgRNA from a *U6:2* promoter and Cas9 mRNA from the *hsp70Bb* promoter (Gokcezade *et al.* 2014). Plasmids were purified (Plasmid Midi Kit; QIAGEN, Hilden, Germany) and dissolved in water.

### Donor template construction

#### pUC-w:

A 2080 bp fragment, spanning genomic nucleotides X:2,792,206–2,790,141 (*D. melanogaster* genome release r6.07), was amplified by PCR from *w^cf^* genomic DNA, sequenced to confirm the *w^cf^* point mutation and identify natural polymorphisms, and inserted into pUC57 between the *Sac*I and *Sph*I sites to produce pUC-*w*. Site-directed mutagenesis was used to mutate the sites targeted by *w* sgRNAs-1, -2, -3, and -4.

#### pUC-armi:

A 2280 bp DNA (synthesized at GenScript, Inc., Piscataway, NJ) spanning genomic nucleotides 3L:3,464,383–3,466,434 was inserted into pUC57 between the *Sac*I and *Sph*I sites. The sequence included silent mutations, a naturally occurring nine-nucleotide deletion polymorphism in *armi* exon 8 that disrupts the *armi* sgRNA-1 target site, a naturally occurring 12-nucleotide deletion polymorphism in the *armi* 3′ UTR, and a 36 nt C-terminal Strep-tag II peptide tag.

#### pCR-zuc:

A 2120 bp PCR fragment spanning genomic nucleotides 2L:11,990,382–11,988,263 was inserted into pCR-Blunt II-TOPO to make pCR-*zuc^WT^*. A 991 bp fragment containing a 3×FLAG peptide tag before the stop codon of *zuc* and silent mutations disrupting four potential sgRNA binding sites were synthesized as a gBlock (Integrated DNA Technologies, Coralville, IA), digested with *Nde*I and *Pac*I, and inserted into pCR-*zuc^WT^* between the *Nde*I and *Pac*I sites to produce pCR-*zuc*.

### Screening for mutations at w

For *armi* targeting, individual injected G0 adults were mated with two *w^1118^*; *+* ; *Dr/TM3*, *Sb* males or virgin females. For *zuc* targeting, *w^1118^*; *Sp/CyO*; *+* was used in place of *w^1118^*; *+* ; *Dr/TM3*, *Sb*. Three- to five-d-old G1 progeny (25°) were assessed by light microscopy (MZ6 Stereomicroscope, Leica Microsystems GmbH, Wetzlar, Germany).

### Screening for mutations at the gene-of-interest

Due to the large number of all-red, white-and-red, and coffee-and-red broods, and their lower chance of harboring gene-of-interest conversion, not all G1 broods were PCR screened. Instead, 44 all-red (37% of total), 46 white-and-red (59%), 8 all-white (100%), 29 coffee-and-red (78%), 11 coffee-and-white (92%), and 15 all-coffee broods (88%) were picked for genotyping. Anesthetized G1 male flies were deposited on a CO_2_ pad, and the 9–10 flies closest to the front edge of the pad were individually mated to corresponding balancer virgin females to generate stocks. After 5 d, the G1 males were removed from the crosses, and 1–3 flies from the same brood were homogenized (Gloor *et al.* 1993) in 30 µl per fly “squishing buffer” [10 mM Tris-Cl, pH 8.0, 1 mM EDTA, 25 mM NaCl, 200 µg/ml freshly diluted Proteinase K solution (AM2546; Thermo Fisher Scientific)] with a plastic pestle (Kimble-Chase Kontes, Vineland, NJ) in 1.7 ml microcentrifuge tubes, incubated at 37° for 30 min, and then the Proteinase K inactivated at 95° for 5 min. PCR was used to amplify 505–1225 bp amplicons spanning the target loci from 1 µl homogenate (15 µl final reaction volume; MeanGreen 2× Taq Master Mix, Empirical Bioscience, Inc., Grand Rapids, MI). We note that using this experimental setup, PCR efficiency drops for amplicons longer than 1 kbp. Because different sgRNAs targeted different regions of *armi* or *zuc*, different PCR primers were designed for each target locus (Table S8 in File S1). Whenever possible, one of the two primers bound only to the genome and not the donor, to avoid amplifying extrachromosomal or ectopically inserted donor DNA. When screening for recombinants with novel sequences knocked-in at the target locus, PCR with one primer bound to the novel sequence (*e.g.*, 3×FLAG) and another primer bound only to the genome and not the donor (Table S8 in File S1) can quickly identify the positive recombinants. When screening for indels or recombinants with point mutations at the target loci, we used the following strategies to identify PCR products that contained such mutations.

#### Restriction enzyme digestion:

Because G1 flies inherit one chromosome from the injected G0 embryo and the other from the balancer fly, at least half of the PCR products were amplified from the wild-type gene. We digested the PCR reaction with a restriction enzyme that cleaves adjacent to the predicted DSB in the wild-type amplicon: PCR products resistant to the restriction digestion should harbor mutations at the recognition site. The uncut PCR product was then gel isolated (QIAquick Gel Extraction Kit, QIAGEN) and sequenced to identify the underlying mutation. This approach ensures that the wild-type PCR products do not confound the sequencing trace and allows the detection of one mutant allele among ≥6 alleles, allowing multiple G1 flies to be pooled in the same PCR. In addition to indels, HR can also be detected by this method, as long as the HR donors are engineered to contain silent mutations that disrupt the restriction enzyme site. A drawback is that the deletion or HR must affect the restriction enzyme recognition sequence; those that do not will remain undetected. The following restriction digestions were used:

armi sgRNA-1 DSB: an *Ava*II site 6 bp away; 5 µl of PCR digested with *Ava*II [0.2 U/µl final concentration (f.c.)] in 0.5× CutSmart Buffer (New England Biolabs, Inc., Ipswich, MA) in 10 µl final volume (f.v.) at 37° for 2 hr;
armi sgRNA-2/3 DSBs: a BstNI site 1 bp (sgRNA-2) or 1 bp (sgRNA-3) away; 5 µl of PCR with *Bst*NI (0.5 U/µl f.c.) in 1× NEBuffer 3.1 (NEB) in 10.5 µl f.v. at 60° for 1 hr;
armi sgRNA-4 DSB: no restriction enzyme site nearby; digested with T7E1 as described below;
armi sgRNA-5/6 DSBs: a PmlI site 17 bp (sgRNA-5) or 11 bp (sgRNA-6) away; 10 µl PCR with *Eco*72I (0.5 U/µl f.c., Thermo Fisher) in 12.5 µl f.v. at room temperature for 1 hr;
zuc sgRNA-1 DSB: a BccI site 9 bp away; 5 µl of PCR with *Bcc*I (0.5 U/µl f.c.) in 0.5× CutSmart Buffer in 10 µl f.v. at 37° for 1 hr;
zuc sgRNA-2 DSB: a *Hpy*CH4III site 7 bp away; 5 µl of PCR with *Hpy*CH4III (0.25 U/µl f.c.) in 0.5× CutSmart Buffer in 10 µl f.v. at 37° for 2 hr.

#### T7 endonuclease I (T7E1) digestion:

To complement the restriction enzyme digestion, the same PCR products were denatured, reannealed to form heteroduplex, and digested with the mismatch-specific, sequence-independent T7E1. In G1 single-fly PCR, either 0% (both alleles are wild-type) or 50% (one allele is mutant) of reannealed products will be substrates for T7E1. The drawback of this approach is: (1) some small sequence changes may escape T7E1 detection (Vouillot *et al.* 2015); (2) lower sensitivity and higher background prevents the pooling of G1 flies in the same PCR; and (3) as the wild-type PCR products cannot be specifically destroyed, the sequencing trace has to be manually inspected to detect a mutation. To digest with T7E1, 5 µl PCR product was denatured at 95° for 5 min, reannealed by reducing the temperature 0.1°/sec to 25° to allow heteroduplex to form, and then digested with T7E1 (0.125 U/µl f.c.) in 1× NEBuffer 2 (NEB) in 10 µl f.v. at 37° for 15 min, as previously described (Zhang *et al.* 2014a).

### Differentiating gene conversion from plasmid integration

The homozygous G3 descendants of the G1 flies carrying HR were further analyzed by PCR to distinguish between gene conversion and plasmid integration. To ensure efficient amplification of PCR amplicons >2 kbp, genomic DNA from G3 homozygotes was isolated by homogenizing 10 flies in 200 µl 2× PK buffer [200 mM Tris-Cl, pH 7.5, 25 mM EDTA, 300 mM NaCl, 2% (w/v) SDS], incubated with 200 µg/ml (f.c.) Proteinase K at 65° for 30 min, extracted with 200 µl buffer-equilibrated phenol:chloroform:isoamyl alcohol (25:24:1 by volume, pH 8.0; AMRESCO LLC, Solon, OH), and centrifuged at 20,800 × *g* for 5 min at room temperature. The top aqueous layer was precipitated with one-tenth volume 3 M sodium acetate and three volumes 100% ethanol on ice for 1 hr. The precipitate was recovered by centrifugation (20,800 × *g* for 15 min at 4°), washed with 70% (v/v) ethanol, air dried, and dissolved in water. To detect gene conversion events, PCR was performed using forward and reverse primers binding exclusively to the genome and the PCR product sequenced to differentiate between gene conversion and plasmid integration. For armi, armi-exon6 forward and CycJ-exon2 reverse primers (Table S8 in File S1) generated a 2539 bp amplicon; for zuc, dgt2-exon2 forward and CG34163-upstream reverse primers generated a 2450 bp amplicon (Phusion DNA Polymerase, NEB; 200 ng genomic DNA, 50 µl reaction volume).

### Statistical analysis

Two-tailed tests were performed using Prism 6 (GraphPad Software, Inc., La Jolla, CA).

### Data availability

Plasmids and fly strains are available upon request. The authors state that all data necessary for confirming the conclusions presented in the article are represented fully within the article.

## Results

### w coconversion facilitates screening for both indels and recombinants

**Figure 1:**
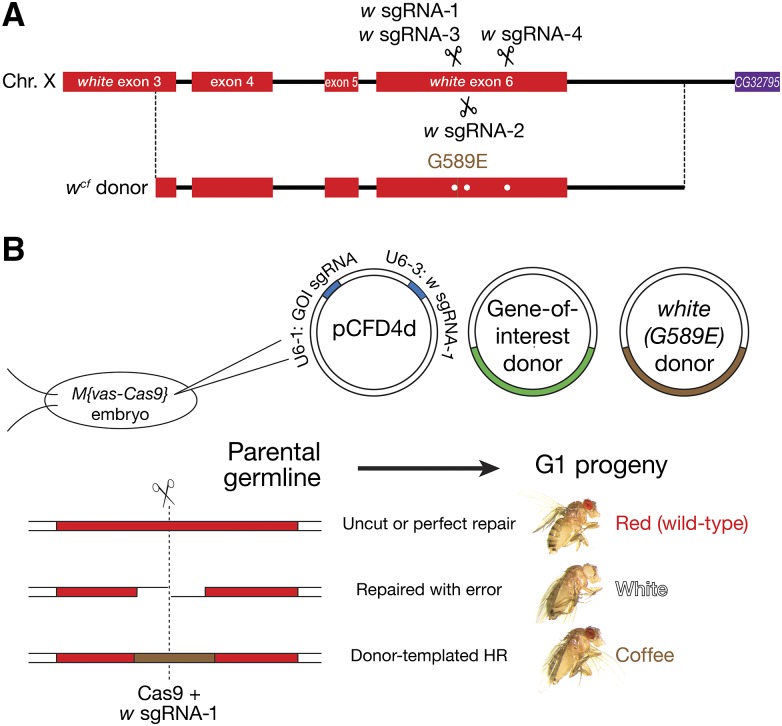
*white* coconversion strategy. (A) The eye pigment gene *white* was cotargeted with the gene-of-interest. The *w^cf^* HR donor carries a GC-to-AA mutation that creates a G589E missense mutation in the White protein. Flies homozygous or hemizygous for *w^cf^* (*i.e.*, *w^cf^/w^1118^* or *w^cf^/*Y) have coffee, instead of the wild-type red, eyes. Scissors mark the target loci of the *white* sgRNAs. Dots on the donor plasmid mark silent mutations that confer resistance to the *white* sgRNAs. (B) Plasmids expressing *w* sgRNA-1 and an sgRNA targeting the gene-of-interest, a plasmid containing the donor for the gene-of-interest (GOI), and a plasmid containing the *w^cf^* donor were coinjected into *Drosophila* syncytial blastoderm embryos that express transgenic Cas9 (*vas*-Cas9). The double-strand break created by *w* sgRNA-1-guided Cas9 may be repaired either perfectly, with nucleotide insertion or deletion (indels), or with sequence copied from the coinjected exogenous donor DNA. The eye color of the G1 progeny reflects the repair mechanism: red eyes indicate perfect repair or no cutting by Cas9; white indicates creation of an indel; and coffee reflects repair by HR.

Changes in eye color are among the most readily identified phenotypes in *Drosophila*. Wild-type eyes are bright red with an obvious pseudopupil. Mutations in *w* generate eye colors ranging from brown to yellow for hypomorphic alleles and white for null alleles. Among the alleles of *w* that are caused by point mutations, *w^coffee^* (*w^cf^*) (Zachar and Bingham 1982) was chosen as the coconversion marker because of its easy-to-screen, reddish brown eyes lacking a pseudopupil. We designed a *w* sgRNA that directs Cas9 to cut 5 bp upstream of the *w^cf^* mutation (*w* sgRNA-1) and an HR donor comprising 2080 bp from the *w^cf^* allele, which differs from wild-type *w* by both a GC-to-AA mutation that creates a G589E missense mutation in the White protein (Mackenzie *et al.* 1999) and silent mutations that confer resistance to the *w* sgRNAs (Figure 1A). HR-mediated repair of the Cas9-catalyzed DSB produces coffee-colored eyes, whereas imprecise end joining generates white eyes when an indel disrupts function of the *w* mRNA or protein. Importantly, ectopic insertion of the HR donor will not produce the coffee-eye phenotype, as the donor carries only 1144 bp of the 2064 bp *w* coding sequence.

To test this strategy, a plasmid containing the *w^cf^* HR donor, a plasmid containing the donor for the gene-of-interest, and a plasmid engineered to express both the *w* sgRNA and an sgRNA targeting the gene-of-interest were coinjected into *Lig4*^+^ or *Lig4*^–^ preblastoderm embryos that express *S. pyogenes* Cas9 (*vas*-Cas9) (Gratz *et al.* 2014). The adult flies that developed from the injected embryos were mated with *w^1118^* flies; the eye colors of the resulting G1 offspring revealed the *w* genotype of the germline stem cells of the G0 parent. The G1 progeny included coffee-, white-, and red-eyed flies (Figure 1B). Sequencing white and coffee G1 flies confirmed that white-eyed flies (*n* = 10/10) had indels at the target site in *w*, whereas flies with coffee-colored eyes contained the G1766A, C1767A *w^cf^* mutation (*n* = 6/6). Thus, eye color provides an effective reporter for *w* sgRNA-directed mutagenesis in the fly germline.

Some G0 produced broods with uniformly red-, white-, or coffee-eyed flies, while others produced broods comprising flies of all possible combinations of the three eye colors. Editing of *w* can occur early in any of the dozens of pole cells that form at the posterior pole of the syncytial blastoderm embryo or later in the descendants of these germ cell progenitors. Because individual G0 pole cells may incorporate different amounts of the injected plasmids, the frequency of DNA cleavage by sgRNA-guided Cas9 and the choice of repair pathways will differ among germ cells, generating variation in the ratio of red-, white-, and coffee-eyed G1 flies. The percentage of nonred G1 flies should reflect the allele frequency of mutant chromosomes in G0 germline stem cells, which in turn reflects the overall targeting efficiency.

**Figure 2:**
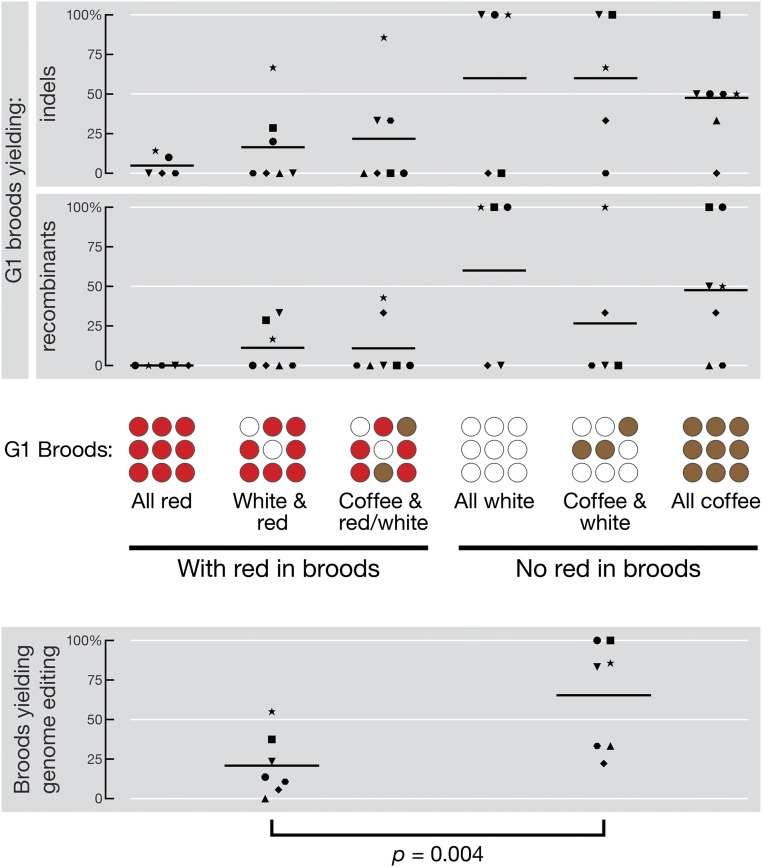
Cooccurrence of *w* and gene-of-interest genomic editing events. Adults from injected G0 embryos that produce G1 broods are either divided into six groups according to their eye color composition: (1) all red; (2) white and red; (3) coffee and red or coffee, white, and red; (4) all white; (5) coffee and white; and (6) all coffee (upper panel), or divided into two categories, “with red in broods” (groups 1–3) and “no red in broods” (groups 4–6; lower panel). For each experiment, the number of broods yielding indels, recombinants (upper panel), or both editing events (lower panel) at the gene-of-interest, as identified by PCR screening of individual G1 progeny, is reported as percentage of total broods sampled. Shapes of data points represent individual experiments described in Table 1. Line presents the mean across all seven experiments. *p*: two-tailed, paired *t*-test.

To test this idea, we assigned each fertile G0 to one of six groups according to the eye color composition of its G1 brood: (1) all red; (2) white and red; (3) all white; (4) coffee and red or coffee, white, and red; (5) coffee and white; and (6) all coffee (Table 1). Six independent experiments cotargeted *w* and *armitage* (*armi*), a third chromosome gene; one experiment cotargeted *w* and *zucchini* (*zuc*), a second chromosome gene. Representative numbers of broods across the six eye color groups were screened by genotyping 9–10 G1 flies from each brood for sequence changes at the gene-of-interest (*i.e.*, *armi* or *zuc*; Table 1). For simplicity, we combined the three groups containing no red-eyed progeny into a single category, “no red in broods,” and the three groups containing at least some red-eyed flies into a single category, “with red in broods.” The fraction of broods that yielded indels or recombinants was 21% ± 19% in the “with red” category, and 65% ± 34% (mean ± SD) in the “no red” category (Figure 2). Therefore, screening for mutations at a gene-of-interest can be restricted to the “no red” broods, which account for 6.3–21% of all broods (mean ± SD = 14% ± 6%, Table 1). For these seven experiments, *w* coconversion would have successfully identified mutants in the gene-of-interest by screening just the 37 “no red” broods (14% of the total 272) using a simple genetic scheme (Figure S1 and *Materials and Methods*).

**Table 1:** Co-targeting *w* and a gene-of-interest.

Shape					Number of G0 whose G1 offspring had eyes that were:					
	G0 *Lig4* genotype	sgRNA plasmid	Donor plasmids	Fertile G0 (*n*)	All red	white & red	Coffee & red/white	All White	Coffee & white	All coffee
•	***Lig4***^***169 a***^	armi-1 & w-1	armi & w	8.9%	10	10	2	1	0	2
		(78 nM)	(132 nM ea.)	(25/281)	EJ: 1/10	EJ: 2/10	EJ: 0/2	EJ: 1/1		EJ: 1/2
					HR: 0/10	HR: 0/10	HR: 0/2	HR: 1/1		HR: 2/2
□	***Lig4***^***169***^	armi-1 & w-1	armi & w	17%	32	7	1	1	1	1
		armi-2 & armi-3	(33 nM ea.)	(43/260)	–	EJ: 2/7	EJ: 0/1	EJ: 0/1	EJ: 1/1	EJ: 1/1
		(26 nM ea.)				HR: 2/7	HR: 0/1	HR: 1/1	HR: 0/1	HR: 1/1
▲	***Lig4***^***+***^	armi-3 & armi-4	armi & w	11%	5	14	2	0	0	4
		w-1 & w-1	(132 nM ea.)	(25/230)	–	EJ: 0/5	EJ: 0/1			EJ: 1/3
		(26 nM ea.)				HR: 0/5	HR: 0/1			HR: 0/3
♦	***Lig4***^***+***^	armi-3 & armi-4	armi & w	20%	27	5	3	3	3	3
		w-1 & w-4	(132 nM ea.)	(44/220)	EJ: 0/13	EJ: 0/2	EJ: 0/3	EJ: 0/3	EJ: 1/3	EJ: 0/3
		(26 nM ea.)			HR: 0/13	HR: 0/2	HR: 1/3	HR: 0/3	HR: 1/3	HR: 1/3
⬣	***Lig4***^***+***^	armi-5 & armi-6	armi & w	29%	31	20	9	0	1	3
		w-1 & w-1	(132 nM ea.)	(64/220)	EJ: 0/9	EJ: 0/10	EJ: 3/9		EJ: 0/1	EJ: 1/2
		(26 nM ea.)			HR: 0/9	HR: 0/10	HR: 0/9		HR: 0/1	HR: 0/2
▼	***Lig4***^***+***^	armi-5 & armi-6	armi & w	17%	7	12	12	1	4	2
		w-1 & w-1	(132 nM ea.)	(38/220)	EJ: 0/5	EJ: 0/6	EJ: 2/6	EJ: 1/1	EJ: 3/3	EJ: 1/2
		(26 nM ea.)			HR: 0/5	HR: 2/6	HR: 0/6	HR: 0/1	HR: 0/3	HR: 1/2
★	***Lig4***^***+***^	zuc-1 & zuc-2	zuc & w	15%	8	10	8	2	3	2
		w-1 & w-1	(132 nM ea.)	(33/222)	EJ: 1/7	EJ: 4/6	EJ: 6/7	EJ: 2/2	EJ: 2/3	EJ: 1/2
		(26 nM ea.)			HR: 0/7	HR: 1/6	HR: 3/7	HR: 2/2	HR: 3/3	HR: 1/2

sgRNA-expressing and HR donor plasmids were coinjected into *Lig4^169^* or *Lig4^+^*, *vas*-Cas9 G0 embryos. Shapes identify the corresponding experiment in Figure 2. *n*, total number of G0 embryos injected, irrespective of fertility or survival. “Coffee & red/white” includes G0 with coffee- and red-eyed, or with coffee-, white-, and red-eyed G1 broods. EJ, broods yielding indels; HR, broods yielding homologous recombinants (plasmid integration and gene conversion); conversion tracts were analyzed only for gene conversion events (Figure 5 and Figure 6).

a Coinjected with 1.2 µM of NLS-Cas9 protein (PNA-Bio, Inc., Thousand Oaks, CA), which had no observable effect.

### Microhomology-mediated end joining is frequent

**Figure 3:**
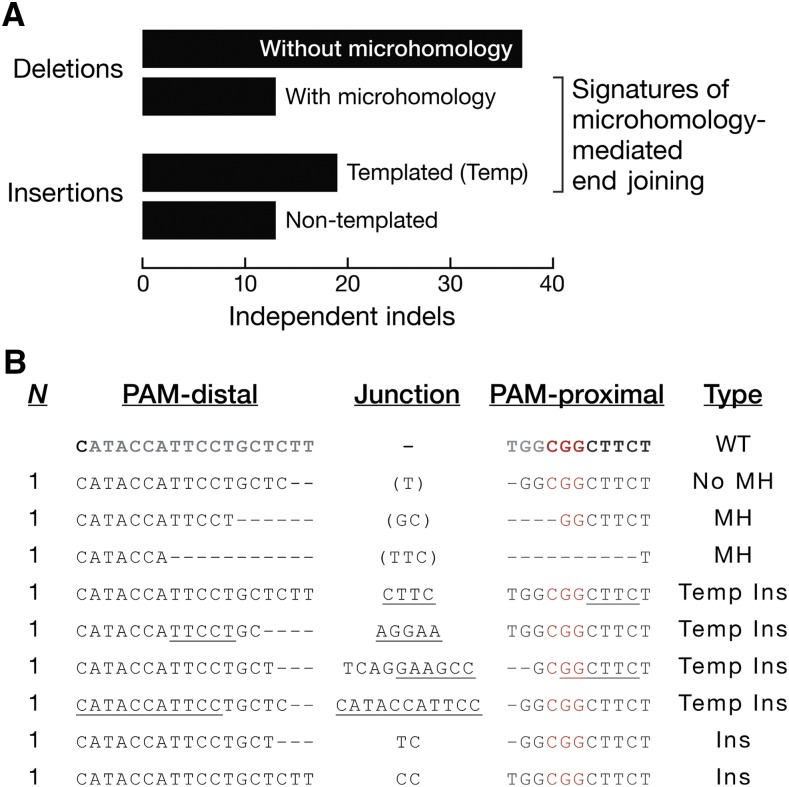
Indel junctional signatures suggest the involvement of microhomology-mediated end joining. (A) Eighty-two independent indels at seven DSBs (Tables S1–S6 in File S1) were classified as deletion without microhomology when there was ≤1 nt of microhomology; as deletion with microhomology when there were ≥2 nt of microhomology; as templated insertion when there were ≥3 nt of inserted nucleotides with identifiable template; or as nontemplated insertion when nucleotide insertions were present without an identifiable template. (B) Indels at the *white* sgRNA-1 target site. The 20 nt sgRNA target sequence is in gray. The PAM sequence is in red. The DSB junction is 3 bp away from the PAM. Dash: deleted nucleotide. Underline: templated insertions at the junction. Nucleotides in parentheses identify microhomologies that can be mapped to either the PAM-distal or PAM-proximal side of the DSB. G0 embryos were *vas-*Cas9, *Lig4^169^*. Ins, nontemplated insertion; MH, deletion with microhomology; *N*, number of independent events; No MH, deletion without microhomology; Temp Ins, templated insertion; WT, wild type.

We identified 82 independent indels at seven sgRNA target sites (Figure 3B and Tables S1–S6 in File S1), and grouped them by ligation junction signatures. Two types of deletions were observed: 13 events showed a pair of ≥2 nt long, identical sequences (microhomology) being reduced to a single sequence via sticky-end ligation; the other 37 events reflected either blunt junctions or only 1 nt of microhomology (Figure 3A). Two types of insertions, often after a deletion, were observed: for 19 events, a sequence ≥3 nt long near the cleavage site appeared to have served as a template for the inserted nucleotides; in the other 13 events, the insertions lacked an obvious template, or were shorter than 3 nucleotides (Figure 3A). Both junctional microhomologies (16% of all events) and templated insertions (23%) are likely products of the microhomology-dependent end-joining pathway, a form of alternative end joining that does not require the canonical nonhomologous end-joining proteins Ku70/80 or Ligase 4 (Chan *et al.* 2010; Yu and McVey 2010; Sfeir and Symington 2015). Consistently, injecting *Lig4^169^* null mutant embryos (McVey *et al.* 2004b) produced microhomologies and templated insertions at *white* or *armi* (Figure 3B and Tables S3 and S4 in File S1).

### A circular plasmid donor frequently integrates at the target locus

**Figure 4:**
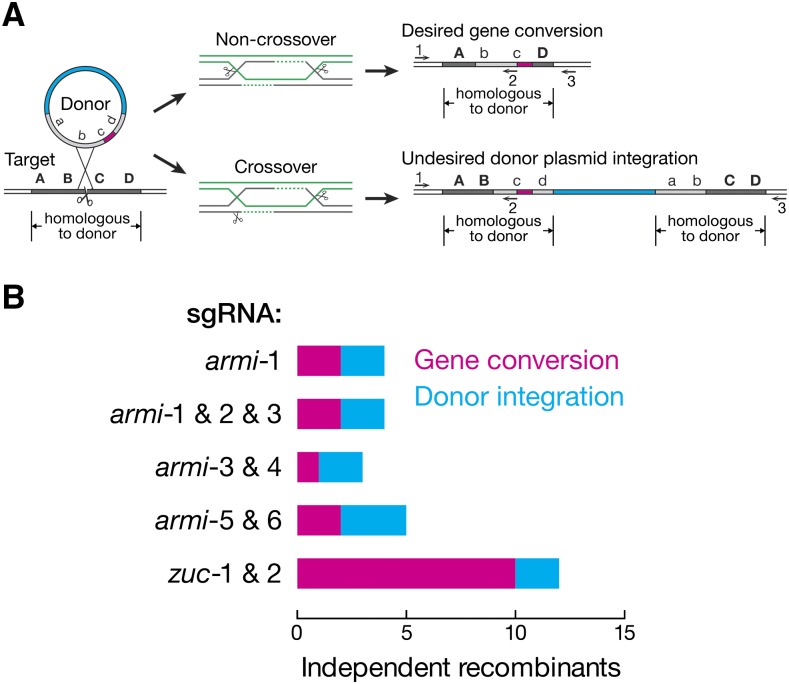
HR using a circular plasmid donor produces either gene conversion or plasmid integration. (A) Two possible outcomes for HR depending on the resolution of double Holliday junctions. PCR primers 1 and 2 can exclude donors present extrachromosomally or ectopically integrated, but cannot differentiate between gene conversion and plasmid integration at the target locus. PCR primers 1 and 3 both bind to the genome and not the donor, allowing unambiguous detection of gene conversion events. (B) Number of gene conversion *vs.* plasmid integration events obtained using different sgRNA combinations.

HR in the gene-of-interest was identified by PCR screening using a primer that binds within both the donor and the genomic locus and a primer that binds exclusively to the genomic sequence. This primer pair can amplify the original or the edited genomic locus, but not donor DNA present extrachromosomally or integrated at an ectopic location. As previously reported (Yu *et al.* 2014), some of the recombinants identified by this strategy corresponded to genomic integration at the gene-of-interest of the entire donor, including the plasmid backbone. In addition to converting the genomic locus to the donor sequence, these recombination events also duplicate the genomic sequence present in the donor (Figure 4A). To distinguish between gene conversion and plasmid integration, we repeated the PCR using primers binding only to the genome and not to sequence present in the HR donor. This strategy readily identified plasmid integration events by their lack of a PCR product or the amplification of a larger-than-expected product. Of the 16 independent HR events identified at *armi*, seven reflected gene conversion while nine integrated the plasmid, a 56% false-positive rate; of the 12 independent HR events identified at *zuc*, 10 underwent gene conversion while 2 integrated the plasmid, a 17% false-positive rate (Figure 4B).

### Gap repair reliably converts the intervening sequence

When gene conversion occurs, the genomic sequence replaced by donor sequence is termed the “conversion tract.” If the conversion tract is short, mutations can only be introduced near the DSB. On the other hand, long conversion tracts allow a single HR event to introduce multiple mutations that are distant from the sgRNA-complementary site. Given that each gene-targeting experiment in *Drosophila* takes 2 to 3 months to accomplish, the ability to introduce two or more edits via a long conversion tract is advantageous. We therefore determined the length of conversion tracts in our experiments.

**Figure 5:**
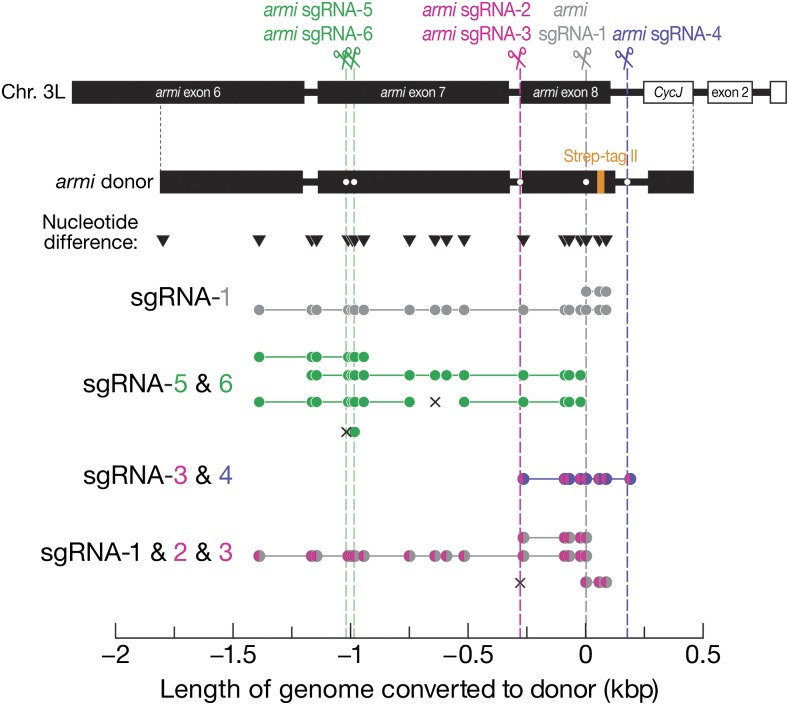
Conversion tracts in *armi* recombinants. The homologous donor carried part of the *armi* gene, a Strep-tag II peptide tag at the end of coding sequence, and 18 sites (inverted triangles) differing in sequence from the endogenous locus that allowed mapping of conversion tracts. Dots on the donor plasmid mark silent mutations that confer resistance to the *armi* sgRNAs. Closed circle, site converted to the donor sequence. Each line presents one recombinant, and the color of closed circles corresponds to the color of the DSB(s) from which HR was initiated (dotted vertical lines). An × indicates an indel.

To introduce a peptide tag at the carboxy terminus of the Armi protein, we assembled a donor plasmid harboring 2280 bp of sequence from the endogenous *armi* locus and introducing a Strep-tag II peptide tag before the stop codon (Figure 5). The donor harbored 19 sites different in sequence from the injected strain, allowing measurement of the length of the conversion tract. We first designed *armi* sgRNA-1 to target a sequence near the end of *armi* exon 8. The HR donor contained 1809 bp upstream and 484 bp downstream of the predicted DSB, and templated two gene conversion events (Table 1). One tract was unidirectional: only the sequence downstream of the DSB (≥77 bp) was converted; the other tract had between 1396 and 1804 bp upstream and ≥77 bp downstream of the DSB converted to the sequence of the donor DNA (Figure 5). We then used two adjacent sgRNAs, sgRNA-5 and -6, targeting sequences in *armi* exon 7. Because the two sgRNA have predicted cleavage sites separated by just 34 bp, we considered them to be a single target site. The same HR donor now contains 790 bp upstream and 1503 bp downstream of the target site, and templated four gene conversion events (Table 1). The first tract converted between 377 and 785 bp upstream and between 68 and 263 bp downstream of the target site; the second between 154 and 377 bp upstream and between 989 and 1068 bp downstream; and the third between 377 and 785 bp upstream and between 989 and 1068 bp downstream (with 37 bp deleted in the middle of the downstream conversion tract). The fourth tract only converted 15 bp upstream of the predicted DSB generated by armi sgRNA-6, and carried a 12 bp deletion at the predicted DSB generated by sgRNA-5, suggesting independent repair events induced by the two guides (Table S6 in File S1). Therefore, conversion tracts initiated from the sgRNA-5/6 target site were unpredictable in directionality and length, just like the *armi* sgRNA-1 site (Figure 5).

In order to more reliably predict the coverage of conversion tracts, we reasoned that by deleting the entire target region, HR could be directed to replace the missing gap using the supplied donor DNA. To achieve this, we targeted armi exon 8 with a pair of guides, sgRNA-3 and -4, whose predicted cleavage sites were separated by 454 bp. The donor includes 1530 bp upstream of the first target site and 286 bp downstream of the second, and templated one gene conversion event (Table 1). As expected when both guides direct Cas9 to cleave the genome, the 454 bp interval between the two DSBs was fully replaced with the sequence contained in the HR template plasmid (Figure 5).

We repeated the same strategy with three sgRNAs whose target sites were separated by 280 bp (sgRNA-1, sgRNA-2, and sgRNA-3; sgRNA-2 and -3 had predicted cleavage sites separated only by 7 bp therefore can be considered as a single target site). The donor included 1530 bp upstream of the first target site and 484 bp downstream of the second, and templated three gene conversion events (Table 1). The first tract reliably replaced the 280 bp gap with that of the donor; the second tract converted between 1117 and 1525 bp upstream of the first target site in addition to a full replacement of the 280 bp gap. The third tract lacked gap repair: the first target site harbored a 2 bp insertion after an 11 bp deletion (Table S4 in File S1); the second site harbored a ≥77 bp conversion tract downstream of the DSB. The 280 bp gap was not converted, suggesting separate repair events at the two target sites.

**Figure 6:**
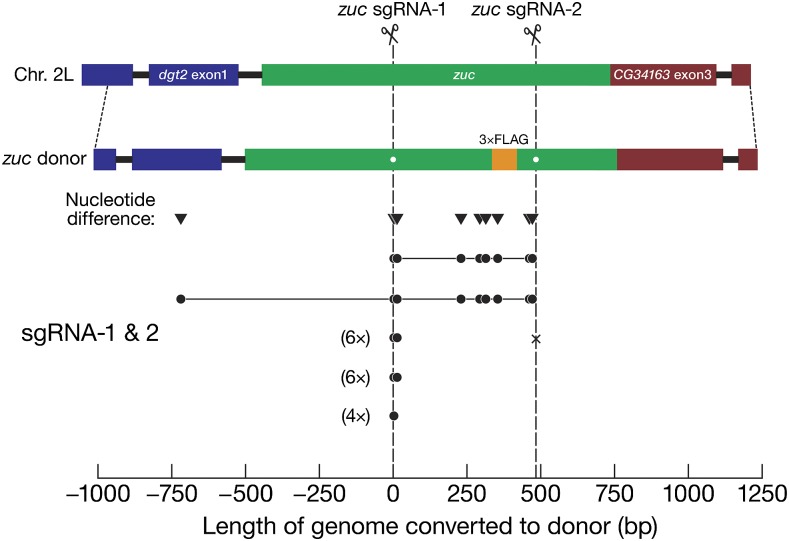
Conversion tracts in *zuc* recombinants. See Figure 5 for details.

We observed a similar gap repair phenomenon when introducing sequence encoding a carboxy terminal 3×FLAG peptide tag into the *zucchini* genomic locus (Table 1 and Figure 6). The two guides, *zuc* sgRNA-1 and -2, targeted sites 395 bp apart. The *zucchini* HR template included 970 bp upstream of the first target site and 760 bp downstream of the second and templated 18 gene conversion events. Of the two gap repair events, one reliably converted the predicted gap, and the other converted ≥720 bp upstream of the first target site in addition to fully replacing the 395 bp gap. The remaining 16 gene conversion events lacked gap repair: only markers near the zuc sgRNA-1 target site were converted. At the zuc sgRNA-2 target site, six contained indels, and ten had wild-type sequence, suggesting separate repair events at the two target sites.

### Ligase 4 mutation does not inhibit end joining or improve HR

In flies, mutation of *Ligase 4* (*Lig4^169^*), a key enzyme in the canonical nonhomologous end-joining pathway, has been proposed to promote HR by suppressing end joining. Zinc-finger nuclease-catalyzed DSBs yield a greater proportion of recombinants in *Lig4^169^* null mutant embryos than in wild-type, but at the cost of decreased fitness of the injected animals (Beumer *et al.* 2008, 2013; Bozas *et al.* 2009). Inhibition of Ligase 4 using RNA interference or small molecule protein inhibitors similarly increased HR efficiency in mosquitos (Basu *et al.* 2015), mice (Maruyama *et al.* 2015), and cultured *Drosophila*, human, or mouse cells (Böttcher *et al.* 2014; Chu *et al.* 2015).

**Figure 7:**
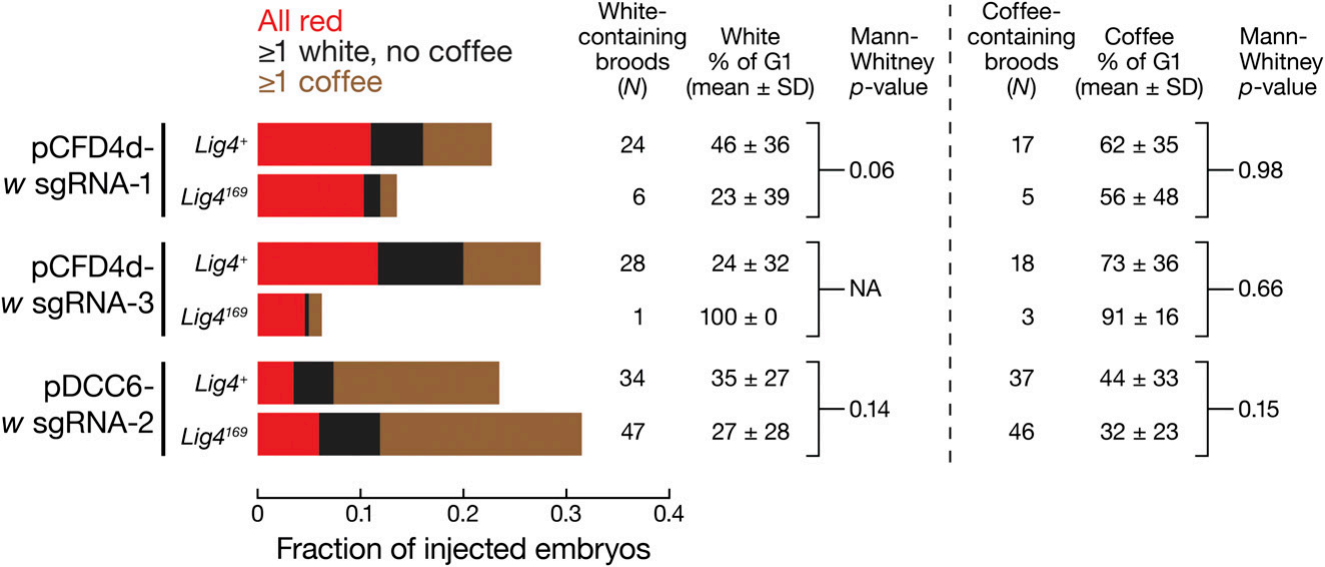
*Lig4^169^* mutant does not inhibit end joining or improve HR. Adults from injected G0 embryos that produce G1 broods were divided into three groups according to their eye color composition: (1) all red; (2) having at least one white, but no coffee, G1; and (3) having at least one coffee G1. For each *w* sgRNA, the percentage of coffee G1 in individual group 3 broods was compared between *Lig4^+^* or *Lig4^169^* embryos. Similarly, the percentage of white G1 in broods with at least one white G1 (with or without coffee G1) was compared. All datasets failed the Shapiro–Wilk normality test, and therefore the two-tailed Mann–Whitney Rank Sum test was used to calculate the *p*-value. NA, *N* was too small to compute a *p*-value.

To test whether *Lig4^169^* null mutants increased the yield of recombinants, we coinjected sgRNA-expressing and HR donor plasmids targeting *w* into *vas-*Cas9, *Lig4^169^* or *vas-*Cas9, *Lig4^+^* embryos. We used the fraction of coffee-producing broods and percentage of coffee-eyed G1 in such broods to score for HR efficiency (Table 2 and Figure 7). Three independent comparisons were conducted, each with a unique sgRNA targeting *white*. *w* sgRNA-1 and sgRNA-3 were provided on the pCFD4d vector together with *armi* sgRNA-1. *w* sgRNA-2 was provided using the pDCC6 vector, which also encodes the Cas9 mRNA (*Materials and Methods*, and Figure 1A). We detected no statistically significant difference between *Lig4^+^* and *Lig4^169^* embryos in producing recombinant, coffee-eyed G1. Similarly, we observed no significant difference between *Lig4^+^* and *Lig4^169^* embryos in producing indels (white-eyed G1, Figure 7). Mothers homozygous for *vas*-Cas9 and either *Lig4^169^* or *Lig4^+^* produce the expected 1:1 Mendelian ratio of red/coffee-eyed or red/white-eyed siblings, excluding the formal possibility that the Cas9-expressing, *Lig4^169^* background affects the recovery of *w* mutant flies. We conclude that the use of *Lig4^169^* embryos does not reduce the recovery of Cas9-induced indels or increase the rate of HR.

**Table 2:** Targeting *w* in *Lig4^+^* or *Lig4^169^*, *vas*-Cas9 G0 embryos

*w* sgRNA	G0 *Lig4* Genotype	Fertile G0 (*n*)	Percent of Fertile G0 Whose G1 Offspring Had Eyes That Were:					
			All Red	White & Red	Coffee & Red/White	All White	Coffee & White	All Coffee
pCFD4d-1 (26 nM)	*Lig4^+^*	23% (255)	48	12	8.6	10	17	3.4
	*Lig4^169^*	14% (310)	76	10	7.1	2.4	0	4.8
pCFD4d-3 (26 nM)	*Lig4^+^*	28% (240)	42	26	6.1	4.5	7.6	14
	*Lig4^169^*	6.3% (240)	73	0	6.7	6.7	0	13
pDCC6-2 (26 nM)	*Lig4^+^*	23% (230)	15	15	52	1.9	7.4	9.3
	*Lig4^169^*	31% (235)	19	18	58	1.4	4.1	0

sgRNA templates were coinjected with 33 nM pUC-w HR donor plasmid DNA. *n*, the number of G0 embryos injected, irrespective of fertility or survival. The coffee & red/white group includes G0 with coffee- and red-eyed, or with coffee-, white-, and red-eyed G1 broods. pCFD4d also carries *armi* sgRNA-1, and pDCC6 also carries a Cas9 gene expression unit.

## Discussion

Our data demonstrate that the coconversion strategy previously used in *C. elegans* (Arribere *et al.* 2014; Kim *et al.* 2014; Ward 2015) can be successfully applied to *Drosophila*, reducing the burden of screening for mutations at the gene-of-interest. The coconversion strategy worked equally well for the generation of indels or recombinants: both types of mutations were enriched in the broods that had no red-eyed progeny (Figure 2 and Table 1). The absence of red-eyed G1 flies in a brood indicates that all germline alleles in the G0 animal underwent targeted genome modification at *w*, reflecting efficient delivery of the guide plasmid to all the pole cells after injection. Our data suggest that when this happens, regardless of the choice of repair pathway, the cotargeted gene-of-interest is more likely to be modified. It is worth noting that Cas9-catalyzed DSBs at *w* and the gene-of-interest were correlated, but we did not observe a correlation between the repair pathways used at *w* and at the gene-of-interest: broods with HR at *w* did not necessarily produce recombinants at the gene-of-interest.

We frequently recovered more than one type of mutation at the gene-of-interest from a single G1 brood, evidence that independent repair events occurred among the dozens of germline stem cells of the G0 founder parent. In other words, the G0 germline is frequently mosaic. As an extreme example, five different indels and three different HR events at *zuc* were identified in the ten G1 flies we genotyped from a brood consisting of 15% white-eyed and 85% coffee-eyed offspring.

At the seven sgRNA target sites we tested, 39% of the 82 independent indels had junctional microhomologies or templated insertions (Figure 3 and Tables S1–S6 in File S1), signatures of the *Lig4*-independent, microhomology-dependent end-joining pathway (Yu and McVey 2010; Sfeir and Symington 2015). We recovered many indels containing such signatures from *Lig4^+^* embryos (Tables S1, S2, S5 and S6 in File S1), suggesting that the microhomology-mediated end-joining pathway normally operates even in the presence of Ligase 4. In fact, *Lig4^169^* mutant embryos produced no fewer indels than *Lig4^+^* embryos (Figure 7), suggesting that a Ligase 4-independent end-joining pathway predominates at generating indels. In *C. elegans*, polymerase θ, but not Lig4, is used to repair Cas9-induced DSBs (van Schendel *et al.* 2015). As in worms, the *Drosophila* polymerase θ (*mus308*) is important for *Lig4*-independent end joining (Chan *et al.* 2010). Future experiments to test whether inactivation of *mus308*, alone or together with *Lig4*, reduces indel mutations in flies are clearly needed.

Eliminating donor integration, in which the plasmid integrates into the target locus instead of promoting the desired gene conversion, remains a challenge for Cas9-targeted HR: in our experiments, such integration accounted for 17–67% (median, 50%) of all HR events (Figure 4). Plasmid integration has been reported to account for 70–100% of Cas9-targeted recombinants and was proposed to reflect the outcome of the resolution of double Holliday junctions formed between the donor and the genome (Figure 4A) (Yu *et al.* 2014). “Ends-in” targeting, in which the circular plasmid donor is linearized *in vivo* using the *I-Sce*I endonuclease to generate DSB in the center of the homologous arm, produced plasmid integration 66% of the time (Rong and Golic 2000). For Cas9-induced HR, the DSB is in the genomic locus instead of the extrachromosomal donor, but is otherwise analogous to “ends-in” targeting. For both, distinguishing gene conversion from plasmid integration is essential.

In theory, a linear donor whose sequence is restricted to the target genomic locus should eliminate the problem of integration. Plasmid donors containing a pair of *w* sgRNA-1 target sites − one before the upstream homology arm and one after the downstream arm, both in the same orientation − are predicted to be cleaved twice by *w* sgRNA-1-guided Cas9, liberating the HR donor from the plasmid DNA. Unfortunately, this donor design was inefficient in producing recombinants in our experiments (data not shown).

Variability in conversion tract length was observed in regions flanking a single DSB or flanking the gap deleted by two concomitant DSBs. Measured from the breaks, some tracts were ∼1000 bp, while others were <50 bp (Figure 5); some were even <7 bp (Figure 6). The conversion of the region flanking the DSB(s) is therefore unpredictable. In contrast, when a pair of sgRNAs was used to direct two DSBs, the intervening sequence was reliably replaced with that of the donor (Figure 5 and Figure 6). Pairs of sgRNAs have been used to change or insert 1–3 kbp of novel sequence into a gene in *Drosophila*, presumably through the same gap repair mechanism (Gratz *et al.* 2014; Ren *et al.* 2014a,b; Yu *et al.* 2014; Zhang *et al.* 2014b; Port *et al.* 2015). Using the sister chromatid as a repair template, gap repair readily restores a 9 kbp gap following *P* element excision (McVey *et al.* 2004a). Alternatively, the conversion of intervening sequence between two DSBs may result from two convergent HR events initiated from each DSB separately. In this scenario, the two DSBs do not have to be created concomitantly. It is worth noting that gap repair does not always happen when two sgRNAs were coinjected, as we frequently observed gene conversion at one target site and either an indel or wild-type sequence at the other (Figure 5 and Figure 6). One possibility is that one of the two sgRNAs was more active than the other, reducing the chance of generating two DSBs at the same time—a prerequisite of gap repair. Thus, it may be prudent to carry out two experiments each using a unique pair of sgRNAs to ensure successful gap repair, which also offers the opportunity to generate two independent recombinants with nonoverlapping potential off-target mutations.

Previous studies with zinc-finger nucleases suggested that *Lig4^169^* mutant embryos promote HR (Beumer *et al.* 2008, 2013; Bozas *et al.* 2009). Surprisingly, the use of *Lig4^169^* embryos did not increase HR efficiency in our experiments (Figure 7), perhaps because Cas9, unlike zinc-finger nucleases, leaves blunt ends (Kim *et al.* 1996; Jinek *et al.* 2012).

In conclusion, cotargeting the *w* gene in *Drosophila* when using Cas9 to alter the fly genome substantially reduces the time and effort required for the molecular identification of mutations in the gene-of-interest. Other organisms with available endogenous or transgenic marker genes should be able to adopt a similar coconversion strategy.

## 

## Supplementary Material

Supplemental Material

## References

[bib1] Arribere J. A. Bell R. T. Fu B. X. Artiles K. L. Hartman P. S., 2014 Efficient marker-free recovery of custom genetic modifications with CRISPR/Cas9 in *Caenorhabditis elegans*. Genetics 198: 837–846. 25161212 10.1534/genetics.114.169730 PMC4224173

[bib2] Baena-Lopez L. A. Alexandre C. Mitchell A. Pasakarnis L. Vincent J. P., 2013 Accelerated homologous recombination and subsequent genome modification in *Drosophila*. Development 140: 4818–4825. 24154526 10.1242/dev.100933 PMC3833436

[bib3] Basu S. Aryan A. Overcash J. M. Samuel G. H. Anderson M. A., 2015 Silencing of end-joining repair for efficient site-specific gene insertion after TALEN/CRISPR mutagenesis in *Aedes aegypti*. Proc. Natl. Acad. Sci. USA 112: 4038–4043. 25775608 10.1073/pnas.1502370112 PMC4386333

[bib4] Beumer K. J. Trautman J. K. Bozas A. Liu J. L. Rutter J., 2008 Efficient gene targeting in *Drosophila* by direct embryo injection with zinc-finger nucleases. Proc. Natl. Acad. Sci. USA 105: 19821–19826. 19064913 10.1073/pnas.0810475105 PMC2604940

[bib5] Beumer K. J. Trautman J. K. Mukherjee K. Carroll D., 2013 Donor DNA utilization during gene targeting with zinc-finger nucleases. G3 (Bethesda) 3: 657–664. 10.1534/g3.112.005439 PMC3618352 23550125

[bib6] Bibikova M. Carroll D. Segal D. J. Trautman J. K. Smith J., 2001 Stimulation of homologous recombination through targeted cleavage by chimeric nucleases. Mol. Cell. Biol. 21: 289–297. 11113203 10.1128/MCB.21.1.289-297.2001 PMC88802

[bib7] Boch J. Scholze H. Schornack S. Landgraf A. Hahn S., 2009 Breaking the code of DNA binding specificity of TAL-type III effectors. Science 326: 1509–1512. 19933107 10.1126/science.1178811

[bib8] Böttcher R. Hollmann M. Merk K. Nitschko V. Obermaier C., 2014 Efficient chromosomal gene modification with CRISPR/*cas9* and PCR-based homologous recombination donors in cultured *Drosophila* cells. Nucleic Acids Res. 42: e89. 24748663 10.1093/nar/gku289 PMC4066747

[bib9] Bozas A. Beumer K. J. Trautman J. K. Carroll D., 2009 Genetic analysis of zinc-finger nuclease-induced gene targeting in Drosophila. Genetics 182: 641–651. 19380480 10.1534/genetics.109.101329 PMC2710147

[bib10] Chan S. H. Yu A. M. Mcvey M., 2010 Dual roles for DNA polymerase theta in alternative end-joining repair of double-strand breaks in Drosophila. PLoS Genet. 6: e1001005. 20617203 10.1371/journal.pgen.1001005 PMC2895639

[bib11] Chen H. M. Huang Y. Pfeiffer B. D. Yao X. Lee T., 2015 An enhanced gene targeting toolkit for *Drosophila*: Golic+. Genetics 199: 683–694. 25555988 10.1534/genetics.114.173716 PMC4349064

[bib12] Christian M. Cermak T. Doyle E. L. Schmidt C. Zhang F., 2010 Targeting DNA double-strand breaks with TAL effector nucleases. Genetics 186: 757–761. 20660643 10.1534/genetics.110.120717 PMC2942870

[bib13] Chu V. T. Weber T. Wefers B. Wurst W. Sander S., 2015 Increasing the efficiency of homology-directed repair for CRISPR-Cas9-induced precise gene editing in mammalian cells. Nat. Biotechnol. 33: 543–548. 25803306 10.1038/nbt.3198

[bib14] Farboud B. Meyer B. J., 2015 Dramatic enhancement of genome editing by CRISPR/Cas9 through improved guide RNA design. Genetics 199: 959–971. 25695951 10.1534/genetics.115.175166 PMC4391549

[bib15] Gloor G. B. Preston C. R. Johnson-Schlitz D. M. Nassif N. A. Phillis R. W., 1993 Type I repressors of P element mobility. Genetics 135: 81–95. 8224830 10.1093/genetics/135.1.81 PMC1205629

[bib16] Gokcezade J. Sienski G. Duchek P., 2014 Efficient CRISPR/Cas9 plasmids for rapid and versatile genome editing in Drosophila. G3 (Bethesda) 4: 2279–2282. 25236734 10.1534/g3.114.014126 PMC4232553

[bib17] Govindan G. Ramalingam S., 2016 Programmable site-specific nucleases for targeted genome engineering in higher eukaryotes. J. Cell. Physiol. 231: 2380–2392. 26945523 10.1002/jcp.25367

[bib18] Gratz S. J. Ukken F. P. Rubinstein C. D. Thiede G. Donohue L. K., 2014 Highly specific and efficient CRISPR/Cas9-catalyzed homology-directed repair in *Drosophila*. Genetics 196: 961–971. 24478335 10.1534/genetics.113.160713 PMC3982687

[bib19] Hsu P. D. Scott D. A. Weinstein J. A. Ran F. A. Konermann S., 2013 DNA targeting specificity of RNA-guided Cas9 nucleases. Nat. Biotechnol. 31: 827–832. 23873081 10.1038/nbt.2647 PMC3969858

[bib20] Hsu P. D. Lander E. S. Zhang F., 2014 Development and applications of CRISPR-Cas9 for genome engineering. Cell 157: 1262–1278. 24906146 10.1016/j.cell.2014.05.010 PMC4343198

[bib21] Jeong J. Y. Yim H. S. Ryu J. Y. Lee H. S. Lee J. H., 2012 One-step sequence- and ligation-independent cloning as a rapid and versatile cloning method for functional genomics studies. Appl. Environ. Microbiol. 78: 5440–5443. 22610439 10.1128/AEM.00844-12 PMC3416421

[bib22] Jinek M. Chylinski K. Fonfara I. Hauer M. Doudna J. A., 2012 A programmable dual-RNA-guided DNA endonuclease in adaptive bacterial immunity. Science 337: 816–821. 22745249 10.1126/science.1225829 PMC6286148

[bib23] Kim H. Ishidate T. Ghanta K. S. Seth M. Conte D., 2014 A co-CRISPR strategy for efficient genome editing in *Caenorhabditis elegans*. Genetics 197: 1069–1080. 24879462 10.1534/genetics.114.166389 PMC4125384

[bib24] Kim Y. G. Cha J. Chandrasegaran S., 1996 Hybrid restriction enzymes: zinc finger fusions to Fok I cleavage domain. Proc. Natl. Acad. Sci. USA 93: 1156–1160. 8577732 10.1073/pnas.93.3.1156 PMC40048

[bib25] Kondo S. Ueda R., 2013 Highly improved gene targeting by germline-specific Cas9 expression in *Drosophila*. Genetics 195: 715–721. 24002648 10.1534/genetics.113.156737 PMC3813859

[bib26] Mackenzie S. M. Brooker M. R. Gill T. R. Cox G. B. Howells A. J., 1999 Mutations in the *white* gene of *Drosophila melanogaster* affecting ABC transporters that determine eye colouration. Biochim. Biophys. Acta 1419: 173–185. 10407069 10.1016/s0005-2736(99)00064-4

[bib27] Maruyama T. Dougan S. K. Truttmann M. C. Bilate A. M. Ingram J. R., 2015 Increasing the efficiency of precise genome editing with CRISPR-Cas9 by inhibition of nonhomologous end joining. Nat. Biotechnol. 33: 538–542. 25798939 10.1038/nbt.3190 PMC4618510

[bib28] McVey M. Adams M. Staeva-Vieira E. Sekelsky J. J., 2004 a Evidence for multiple cycles of strand invasion during repair of double-strand gaps in Drosophila. Genetics 167: 699–705. 15238522 10.1534/genetics.103.025411 PMC1470890

[bib29] McVey M. Radut D. Sekelsky J. J., 2004 b End-joining repair of double-strand breaks in *Drosophila melanogaster* is largely DNA ligase IV independent. Genetics 168: 2067–2076. 15611176 10.1534/genetics.104.033902 PMC1448732

[bib30] Moscou M. J. Bogdanove A. J., 2009 A simple cipher governs DNA recognition by TAL effectors. Science 326: 1501. 19933106 10.1126/science.1178817

[bib31] Port F. Chen H. M. Lee T. Bullock S. L., 2014 Optimized CRISPR/Cas tools for efficient germline and somatic genome engineering in *Drosophila*. Proc. Natl. Acad. Sci. USA 111: E2967–E2976. 25002478 10.1073/pnas.1405500111 PMC4115528

[bib32] Port F. Muschalik N. Bullock S. L., 2015 Systematic evaluation of *Drosophila* CRISPR tools reveals safe and robust alternatives to autonomous gene drives in basic research. G3 (Bethesda) 5: 1493–1502. 25999583 10.1534/g3.115.019083 PMC4502383

[bib33] Qi L. S. Larson M. H. Gilbert L. A. Doudna J. A. Weissman J. S., 2013 Repurposing CRISPR as an RNA-guided platform for sequence-specific control of gene expression. Cell 152: 1173–1183. 23452860 10.1016/j.cell.2013.02.022 PMC3664290

[bib34] Ren X. Yang Z. Mao D. Chang Z. Qiao H. H., 2014 a Performance of the Cas9 nickase system in Drosophila melanogaster. G3 (Bethesda) 4: 1955–1962. 25128437 10.1534/g3.114.013821 PMC4199701

[bib35] Ren X. Yang Z. Xu J. Sun J. Mao D., 2014 b Enhanced specificity and efficiency of the CRISPR/Cas9 system with optimized sgRNA parameters in *Drosophila*. Cell Reports 9: 1151–1162. 25437567 10.1016/j.celrep.2014.09.044 PMC4250831

[bib36] Rong Y. S. Golic K. G., 2000 Gene targeting by homologous recombination in *Drosophila*. Science 288: 2013–2018. 10856208 10.1126/science.288.5473.2013

[bib37] Sander J. D. Joung J. K., 2014 CRISPR-Cas systems for editing, regulating and targeting genomes. Nat. Biotechnol. 32: 347–355. 24584096 10.1038/nbt.2842 PMC4022601

[bib38] Sfeir A. Symington L. S., 2015 Microhomology-mediated end joining: a back-up survival mechanism or dedicated pathway. Trends Biochem. Sci. 40: 701–714. 26439531 10.1016/j.tibs.2015.08.006 PMC4638128

[bib39] Smith J. Berg J. M. Chandrasegaran S., 1999 A detailed study of the substrate specificity of a chimeric restriction enzyme. Nucleic Acids Res. 27: 674–681. 9862996 10.1093/nar/27.2.674 PMC148231

[bib40] Sternberg S. H. Doudna J. A., 2015 Expanding the biologist’s toolkit with CRISPR-Cas9. Mol. Cell 58: 568–574. 26000842 10.1016/j.molcel.2015.02.032

[bib41] van Schendel R. Roerink S. F. Portegijs V. Van Den Heuvel S. Tijsterman M., 2015 Polymerase θ is a key driver of genome evolution and of CRISPR/Cas9-mediated mutagenesis. Nat. Commun. 6: 7394. 26077599 10.1038/ncomms8394 PMC4490562

[bib42] Vouillot L. Thélie A. Pollet N., 2015 Comparison of T7E1 and surveyor mismatch cleavage assays to detect mutations triggered by engineered nucleases. G3 (Bethesda) 5: 407–415. 25566793 10.1534/g3.114.015834 PMC4349094

[bib43] Ward J. D., 2015 Rapid and precise engineering of the *Caenorhabditis elegans* genome with lethal mutation co-conversion and inactivation of NHEJ repair. Genetics 199: 363–377. 25491644 10.1534/genetics.114.172361 PMC4317648

[bib44] Yu A. M. McVey M., 2010 Synthesis-dependent microhomology-mediated end joining accounts for multiple types of repair junctions. Nucleic Acids Res. 38: 5706–5717. 20460465 10.1093/nar/gkq379 PMC2943611

[bib45] Yu Z. Chen H. Liu J. Zhang H. Yan Y., 2014 Various applications of TALEN- and CRISPR/Cas9-mediated homologous recombination to modify the *Drosophila* genome. Biol. Open 3: 271–280. 24659249 10.1242/bio.20147682 PMC3988796

[bib46] Zachar Z. Bingham P. M., 1982 Regulation of *white* locus expression: the structure of mutant alleles at the *white* locus of Drosophila melanogaster. Cell 30: 529–541. 6291773 10.1016/0092-8674(82)90250-1

[bib47] Zhang X. Ferreira I. R. Schnorrer F., 2014 a A simple TALEN-based protocol for efficient genome-editing in *Drosophila*. Methods 69: 32–37. 24680697 10.1016/j.ymeth.2014.03.020

[bib48] Zhang X. Koolhaas W. H. Schnorrer F., 2014 b A versatile two-step CRISPR- and RMCE-based strategy for efficient genome engineering in Drosophila. G3 (Bethesda) 4: 2409–2418. 25324299 10.1534/g3.114.013979 PMC4267936

