# Supplementary material for: Rapid Screening for CRISPR-Directed Editing of the Drosophila Genome Using white Coconversion
Source: G3 (Bethesda). 2016 Aug 19;6(10):3197–206. doi: 10.1534/g3.116.032557 (PMC5068941; doi:10.1534/g3.116.032557)
Supplement: Supplemental Material [file supp_6_10_3197__index.html]

Rapid Screening for CRISPR-Directed Editing of the Drosophila Genome Using white Co-Conversion — Rapid Screening for CRISPR-Directed Editing of the Drosophila Genome Using white Co-conversion — Rapid Screening for CRISPR-Directed Editing of the Drosophila Genome Using white Coconversion — Supplemental Material 

# Rapid Screening for CRISPR-Directed Editing of the *Drosophila* Genome Using *white* Coconversion

## Supplemental Material for Ge *et al.*, 2016

**Files in this Data Supplement:**

- Figure S1 - Genetic scheme used to screen and establish CRISPR-edited stocks. (.pdf, 1,383 KB)
- Supplemental Tables - This file contains all Supplemental Tables. (.xlsx, 53 KB)
