# Supplementary figures and images for: Rapid Screening for CRISPR-Directed Editing of the Drosophila Genome Using white Coconversion
Source: G3 (Bethesda). 2016 Aug 19;6(10):3197–206. doi: 10.1534/g3.116.032557 (PMC5068941; doi:10.1534/g3.116.032557)

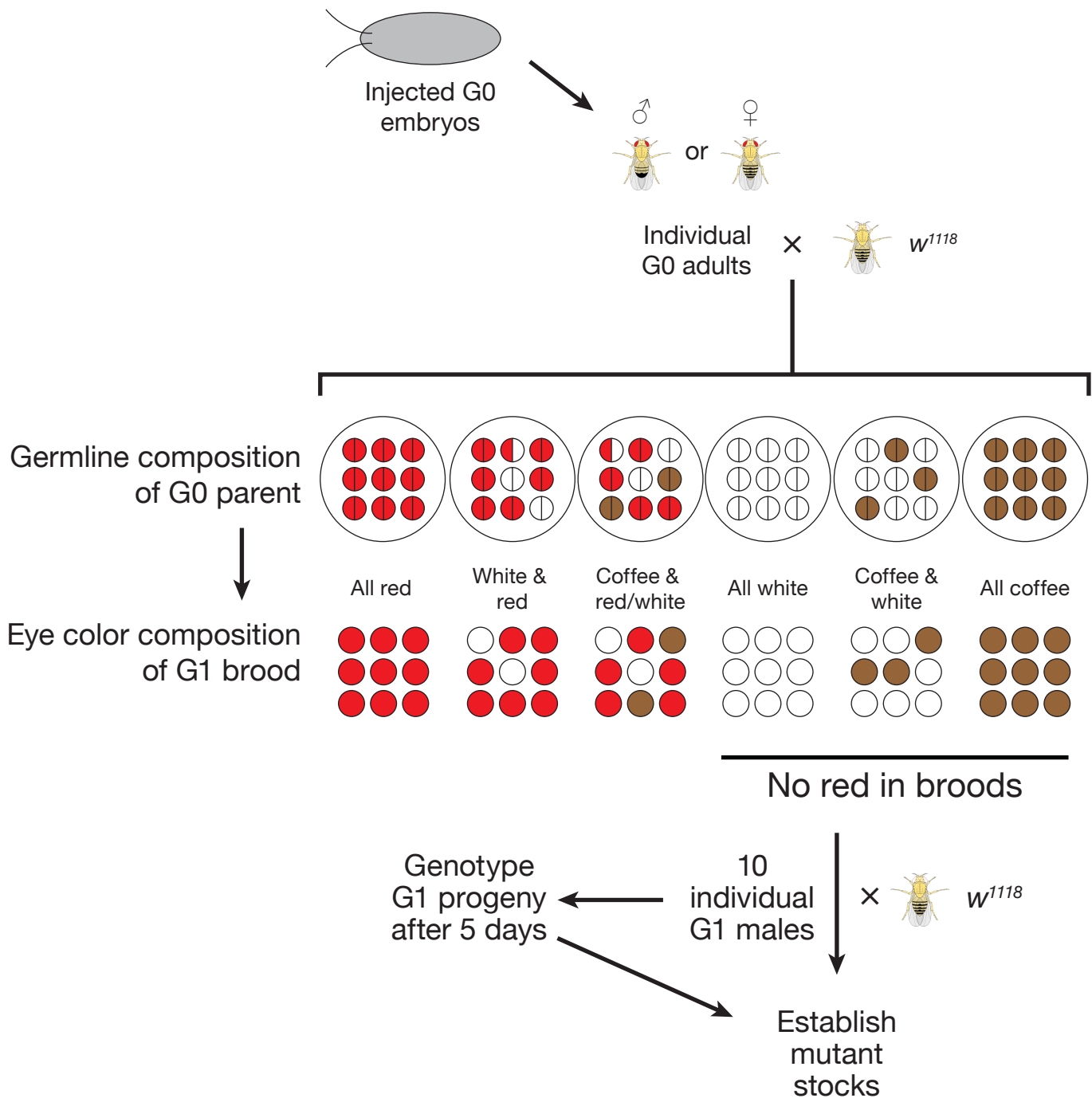

**Figure S1.** Genetic scheme used to screen and establish CRISPR-edited stocks.

Supplement: Supplemental Material [file supp_g3.116.032557_FigureS1.pdf]
